# Respective implications of glutamate decarboxylase antibodies in stiff person syndrome and cerebellar ataxia

**DOI:** 10.1186/1750-1172-6-3

**Published:** 2011-02-04

**Authors:** Mario U Manto, Christiane S Hampe, Véronique Rogemond, Jérome Honnorat

**Affiliations:** 1 Laboratoire de Neurologie Expérimentale, Hôpital Erasme, ULB, Bruxelles, Belgium; 2 Department of Medicine, Division of Metabolism, Endocrinology and Nutrition, University of Washington, Seattle, USA; 3 Hospices Civils de Lyon, Hôpital Neurologique, Centre de Référence Maladie Rare "Syndromes neurologiques Paranéoplasiques", Neurologie B, F-69677 Bron, France; 4 INSERM, U842, Lyon, F-69372 France; Université de Lyon, Lyon1, UMR-S842 Lyon, F-69003 France

## Abstract

**Background:**

To investigate whether Stiff-person syndrome (SPS) and cerebellar ataxia (CA) are associated with distinct GAD65-Ab epitope specificities and neuronal effects.

**Methods:**

Purified GAD65-Ab from neurological patients and monoclonal GAD65-Ab with distinct epitope specificities (b78 and b96.11) were administered in vivo to rat cerebellum. Effects of intra-cerebellar administration of GAD65-Ab were determined using neurophysiological and neurochemical methods.

**Results:**

Intra-cerebellar administration of GAD65-Ab from a SPS patient (Ab SPS) impaired the NMDA-mediated turnover of glutamate, but had no effect on NMDA-mediated turnover of glycerol. By contrast, GAD65-Ab from a patient with cerebellar ataxia (Ab CA) markedly decreased the NMDA-mediated turnover of glycerol. Both GAD65-Ab increased the excitability of the spinal cord, as assessed by the F wave/M wave ratios. The administration of BFA, an inhibitor of the recycling of vesicles, followed by high-frequency stimulation of the cerebellum, severely impaired the cerebello-cortical inhibition only when Ab CA was used. Moreover, administration of transcranial direct current stimulation (tDCS) of the motor cortex revealed a strong disinhibition of the motor cortex with Ab CA. Monoclonal antibodies b78 and b96.11 showed distinct effects, with greater effects of b78 in terms of increase of glutamate concentrations, impairment of the adaptation of the motor cortex to repetitive peripheral stimulation, disinhibition of the motor cortex following tDCS, and increase of the F/M ratios. Ab SPS shared antibody characteristics with b78, both in epitope recognition and ability to inhibit enzyme activity, while Ab CA had no effect on GAD65 enzyme activity.

**Conclusions:**

These results suggest that, in vivo, neurological impairments caused by GAD65-Ab could vary according to epitope specificities. These results could explain the different neurological syndromes observed in patients with GAD65-Ab.

## Background

Stiff person syndrome (SPS) is a rare neurological disease with features of an autoimmune disease. It is characterized by progressive muscle stiffness, trigger-induced spasms, spinal deformity, and high affinity autoantibodies to the smaller isoform of glutamate decarboxylase (GAD65-Ab) [1]. GAD65-Ab are also found in other immune-mediated disorders affecting the central nervous system (CNS), including some patients with cerebellar ataxia (CA) [2,3], and in the majority of patients with autoimmune type 1 diabetes (T1D) [4]. While in T1 D GAD65-Ab are mostly considered as indicators of islet autoimmunity, in SPS a pathogenic role of GAD65-Ab has been postulated based on the finding that they inhibit the enzyme activity of GAD65 in vitro [5,6], and their potential interference with GAD65-mediated transport of GABA-containing vesicles to the presynapse [7,8], both of which may lead to the reduced GABA levels detected in cerebrospinal fluid and brain of SPS patients [9].

A direct role of GAD65-Ab in the pathogenesis of neurological disorders has been questioned because of the assumed impermeability of neurons to immunoglobulins. However, recent work demonstrated that antibodies can be internalized by neurons including Purkinje cells, enabling the antibodies to bind intracellular antigens [10,11].

We previously demonstrated that IgG purified from GAD65-Ab positive patients with neurological syndromes impair cerebellar activity and learning, and affect spinal cord activity in rodents [12]. First, we assessed the increase in the cortical motor response normally associated with repeated somatosensory stimulation in rodents, an effect mediated by the cerebellum, which is considered as a first step of learning in the paradigm of sustained peripheral stimulation [13-15]. Administration of IgG isolated from GAD65-Ab positive neurological patients induced repetitive muscle discharges, abnormal exteroceptive reflexes and increased F/M ratios, suggesting IgG-enhanced motoneuronal excitability. Second, IgG isolated from GAD65-Ab positive neurological patients significantly impaired the synaptic regulation of glutamate after N-methyl-D-aspartate (NMDA) administration. IgG from GAD65-Ab positive individuals without CNS involvement were ineffective in both models. Recently, Sommer et al. reported that injections of rats with the IgG fraction of an SPS patient with anti-amphiphysin antibodies resulted in a dose-dependent stiffness with spasms mimicking those of human SPS [16,17]. Taken together, these results strongly support that SPS is directly caused by the effect of antibodies on spinal cord neurons, both in anti-amphiphysin and GAD65-Ab positive cases. However, IgG from GAD65-Ab positive SPS patients and CA patients caused the same types of dysfunction in the cerebellum and in the spinal cord, leaving unexplained why these patients typically develop distinct clinical pictures, although some patients exhibit both syndromes [18-20]. While immunotherapy and IgG-depleting strategies often alleviate symptoms of GAD65-Ab positive SPS, symptoms of cerebellar dysfunction rarely improve [20-22]. A possible explanation for this observation may be distinct differences in the cascade of events induced by antibodies and differences in the vulnerability of various sites in the CNS to GAD65-Ab. GAD65-Ab acting upon cerebellar pathways might induce lesions reaching an irreversible stage, with neuronal destruction and cerebellar atrophy in a chronic situation. This hypothesis is supported by the recent publication of an autopsy of a patient with both CA and SPS showing only Purkinje cells loss and no abnormalities in the spinal cord [19].

In the present study, we used IgG from GAD65-Ab positive patients exhibiting CA or SPS and found differences between both diseases in the glycerol turnover, an indicator of the turnover of cellular membranes. These differences were enhanced by Brefeldin-A (BFA), an inhibitor of the recycling of vesicles [23,24], when high-frequency stimulation of the cerebellum, a depleting procedure of vesicles, was applied. In addition, this procedure revealed differences in terms of cerebellocortical inhibition and F/M ratios. This suggested that IgG from GAD65-Ab-positive patients exert disease-specific levels of impairment, possibly caused by different GAD65-Ab epitope specificities. We tested this hypothesis using two monoclonal GAD65-Ab with distinct epitope specificities, of which only one (b78) inhibits GAD65 enzyme activity [25].

## Methods

### Antibodies

#### Polyclonal serum IgG isolated from GAD65-Ab positive individuals

Sera were collected from a GAD65-Ab positive SPS patient (Ab SPS) and a GAD65-Ab positive CA patient (Ab CA). Control sera were obtained from a patient with encephalitis (Ab Ctrl). None of the patients had T1 D. All sera were stored at -80°C. Experiments were performed with IgG purified on protein A-Sepharose (protein A Sepharose 4 fast flow, Amersham Biosciences, France). IgG were dialyzed overnight at 4°C against Ringer's solution (Frenesius Kabi, France) and sterilized by filtration with 0.22 μm filters. Protein concentrations were adjusted to 2.5 mg/ml. Five μl of the purified antibody (12.5 μg) were injected per experiment.

#### Monoclonal antibodies b78 and b96.11

Human monoclonal antibodies b96.11 and b78 specific to GAD65 were derived from a patient with Autoimmune Polyendocrine Syndrome Type 1 [26], and isolated from supernatants of the respective B cell lines. The protein concentration was adjusted to 1 mg/ml. Five μl of the purified antibody (5 μg) were injected per experiment.

The conformational epitope recognized by b96.11 is bound by the majority of GAD65-Ab of T1 D patients [27] and less frequently by GAD65-Ab present in patients with SPS [25]. The conformational epitope recognized by b78 is rarely bound by GAD65-Ab of T1 D patients, and is associated with GAD65-Ab in SPS patients [25]. Notably, only b78 inhibits the enzyme activity of GAD65 [25].

### GAD65Ab epitope mapping

Recombinant [^35^S]GAD65 was produced in an in vitro-coupled transcription/translation system with SP6 RNA polymerase and nuclease-treated rabbit reticulocyte lysate (Promega, Madison, WI, USA) as described previously [28,29]. The in vitro-translated [^35^S]GAD65 was kept at -70°C and used within 2 wk. The capacity of the recombinant Fab (rFab) of the above described GAD65-specific monoclonal antibodies to compete with serum GAD65-Ab for binding to [^35^S]GAD65 was tested in a competitive Radioligand binding assay (RBA) using protein A-Sepharose (Invitrogen, Carlsbad, CA, USA) as the precipitating agent [27]. Significant reduction in binding was determined as less than 80% remaining binding through the use of rFab D1.3 specific to Hen Egg Lysozyme.

### GAD65 enzyme activity assay

GAD65 activity was measured by the ^14^CO_2_-trapping method described previously [30]. Recombinant human GAD65 (100 ng) was incubated with reaction buffer (50 mM K_2_HPO_4_, 0.03 mM PLP, and 0.1 mM DTT (pH 6.8)) for 1 h at room temperature with or without serum (5-15 μl). The enzymatic reaction was initiated by the addition of 0.56 mM L-glutamate and 0.018 μCi of ^14^C-glutamate (Perkin Elmer, Waltham, MA, USA) and allowed to continue for 2 h at 37°C with gentle agitation. During incubation, released ^14^CO_2 _was captured on filter paper (Kontes Vineland, NJ, USA) soaked in 50 μl of 1 M NaOH. After the incubation, the absorbed radioactivity was determined in a Beckman scintillation counter. The results are presented as follows: percentage of residual activity = counts per minute in the presence of serum/counts per minute in the absence of serum × 100.

### Animal studies

Animal studies were approved by the institutional animal care committee of the Free University of Brussels. Experiments were conducted in male Wistar rats (Charles River Laboratories; weight between 240 and 430 g). See Table 1 for a summary of the experiments carried out.

**Table 1:** Summary of experiments carried out

Experiments	Purified IgG fractions/Control solutions	Purified IgG fractions/GAD65-Ab
**^a^Modulation of motor cortex excitability**	No injection in 7 rats	b78 in 4 rats
	Ringer's in 7 rats	b96.11 in 4 rats
**^b^Cerebello-cortical inhibition**	Ab Ctrl in 4 rats	Ab CA in 3 rats
		Ab SPS in 3 rats
**^c^F/M ratios**	Ab Ctrl in 4 rats	Ab CA in 3 rats
	No injection in 6 rats	Ab SPS in 3 rats
	Ringer's in 6 rats	b78 in 6 rats
		b96.11 in 6 rats

**^a^**Study of the modulation of the excitability of the motor cortex following repetitive stimulation of the contralateral sciatic nerve;

**^b^**Cerebello-cortical inhibition following high-frequency stimulation of the cerebellar cortex and following transcranial direct current stimulation (tDCS)

^c^Study of the ratios F waves/M response following stimulation of the left tibial nerve and recording in the plantaris muscle

#### Microdialysis procedure

**Figure 1:**
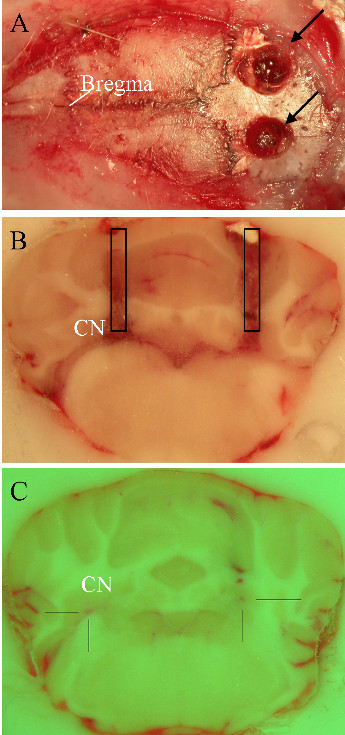
**Illustration of bilateral implantation of cerebellar guides**. A: Two holes (black arrows) in the skull to implant the guides. B: Probes are inserted in cerebellar nuclei (CN). C: Bilateral injection of Ab. Brain sections at the level of cerebellar nuclei in B and C.

This method has been explained earlier [12]. Briefly, anesthetized animals were fixed in a stereotaxic apparatus (accuracy < 0.1 mm). The head was leveled and secured by ear bars and a tooth holder. The skull was exposed, scalp was shaved, cut sagitally and tissues overlying the cranium were removed. An intra-cerebellar guide was implanted and fixed (coordinates of the extremity of the guide were AP: -11.6, L: +2.3, V: -4.6; all coordinates are related to bregma according to the atlas of Paxinos and Watson) (Figure 1). A CMA/10 probe (length: 2 mm, diameter: 0.5 mm) was inserted in the cerebellum. The probe was connected to a microinfusion pump and perfused with Ringer's at a flow rate of 2 μL/min. A volume of 5 μL of solution was injected over a period of 5 minutes through the guide. The probe was then reinserted in the guide (CMA12, Carnegie Medicin AB, Sweden) and continuously perfused with Ringer's at a flow rate of 2 μL/min. All animals underwent cerebellar surgery on the left side. At the end of the experiments, an overdose of chloral hydrate was administered and the brain was removed for histological verification of the location of the probe [12]. Experiments with misplacements of the probes were excluded. For experiments with administration of BFA, a bilateral targeting of cerebellar nuclei procedure was applied. For these experiments, each rat received the Ab followed by BFA on one side, and the Ab followed by Ringer's contralaterally.

#### Glutamate, glucose, lactate, pyruvate and glycerol measurements

After stabilization of baseline measurements, samples were collected every 20 minutes to measure the extracellular concentrations of glutamate, glucose, lactate, pyruvate and glycerol. We used the CMA/600 device (Aurora Borealis Control; glutamate oxidase method) for the quantitative determination of metabolites in microdialysates [31].

#### Mapping of corticospinal responses

Stimuli (square wave of 1 msec duration) were first applied every mm in the sagittal axis and every 0.5 mm in the coronal axis to obtain a matrix of 6 × 9 sites of stimulation, [32] to identify the "hot spot" corresponding to the largest motor evoked potential. We subsequently used the location of 3 mm laterally and 0.5 mm posterior to bregma for stimulation of the motor cortex, consistent with stimulation sites in other studies [14,33]. Recruitment curves (detection of motor threshold MT defined as the lowest intensity eliciting at least 5 out of 10 evoked responses with an amplitude >20 μVolts, followed by increases of the intensity of stimulation with steps of 0.1 mAmp until maximum) of corticomotor responses were analyzed to confirm the classical sigmoid course [34]. Motor cortex was stimulated at an intensity of 130% of MT to assess latencies and amplitudes of corticomotor potentials.

#### Modulation of motor cortex excitability following peripheral repetitive stimulation

We investigated the motor evoked responses (MEPs: motor evoked potentials) in the left gastrocnemius muscle following stimulation of the contralateral motor cortex, before (basal condition) and after repetitive electrical stimulation of the left sciatic nerve [13,14]. Chloral hydrate was administered continuously at 2 μL/min (CMA100 micropump; CMA, Sweden) using the i.p. route [2]. Anesthesia depth was adjusted for absence of abdominal contractions in response to tail pinch. The left sciatic nerve was surgically exposed for bipolar stimulation. Duration of stimulation was one hour. Trains of stimulation were delivered at a rate of 10 Hz (a train being composed of 5 stimuli of a 1 msec duration; A310-A365 stimulator - World Precision Instruments, UK). Stimulus intensity was adjusted to produce constant somatosensory evoked potentials (SEP) in the EEG [14]. For the stimulation of the motor cortex, square stimuli were applied at the level of the motor cortex [15]. Peak-to-peak amplitudes in motor responses of the contralateral gastrocnemius muscle were studied. Filter settings were 30 Hz-1.5 KHz (NeuroMax 4, Xltek, Canada).

#### Transcranial direct current stimulation (tDCS)

The methodology is described elsewhere [32]. Corticomotor responses evoked in the gastrocnemius muscle following stimulation of the contralateral motor cortex, before (basal condition) and after application of trains of tDCS were studied. The amplitudes of corticomotor responses were also studied in the contralateral side (gastrocnemius muscle) before and after tDCS over left/right motor cortex (random selection of side of stimulation was applied). Peak-to-peak amplitudes in motor responses of the left (right) gastrocnemius muscle were studied before and after tDCS for 10 corticomotor responses. We used subcutaneous electrodes (Technomed 017K025) implanted in muscles. We obtained similar results by folding wires (Wire silver, AGT0510, World Precision Instruments) into flat plates implanted into a subcutaneous pocket over the gastrocnemius muscle [14]. Trains of electrical stimuli were applied on the skull with the anode placed at the level of the right (left) motor cortex, just anteriorly to the site of stimulation used to obtain corticomotor responses. For anodal stimulation, we followed the protocol by Fregni et al. to obtain a contact area of 7.1 mm^2 ^[35]. The cathode (diameter 0.8 mm) was placed ipsilaterally on the supraorbital region (inter-electrode distance: 5 mm). Duration of stimulation was 20 minutes [36] (a duration of stimulation of 7 minutes at 1 mA is known to induce significant changes of motor cortical excitability in human). Stimulus intensity was 0.4 mAmp. tDCS was applied directly onto the cranium to ensure a defined contact area over the cortex.

#### Cerebello-cortical inhibition

Stimulation of the cerebellum inhibits motor cortex activity [37]. To assess the cerebello-cortical inhibition, the dura around the cerebellum was exposed and the insertion of cervical muscles was cut to avoid the afferent volley from these muscles. An electrical stimulus was applied over the cerebellum (4 concomitant sites of stimulation; A310-A365 stimulator) followed by a second stimulus applied contralateral over the motor cortex after 2.1 msec delay. This delay was first determined according to the inhibition/inter-stimulus interval (ISI) curve determined in 3 rats. Measurement of cerebello-cortical inhibition was performed after administration of antibodies, following high frequency stimulation of the cerebellum, and following applications of trains of tDCS. Each duo of conditioned/unconditioned response was assessed 10 times to compute the average.

#### F-waves and M response

The F-waves and the direct motor responses (M response) were studied as described [38]. Electrical stimulation of the left tibial nerve was achieved through a pair of needle electrodes inserted subcutaneously at the ankle, behind the medial malleolus. Electrical stimuli consisted of single square-wave shocks of 0.5 msec duration, delivered every 6 seconds. EMG recordings were obtained from the ispilateral plantaris muscle through a pair of needle electrodes inserted in the distal third of the sole (filters 30 Hz-1.5 KHz). We assessed the ratio mean F/mean M wave amplitudes following 50 supramaximal stimuli [13,39]. We analyzed these ratios after administration of antibodies, following high-frequency stimulation of the cerebellar cortex, and following application of trains of tDCS.

### Statistical analysis

The levels of glutamate, glucose, lactate/pyruvate ratios, and glycerol levels during infusion of NMDA were compared with the analysis of variance. The effects of Ab on the cerebello-cortical inhibition were compared using the analysis of variance, followed by multiple comparison procedure (Holm-Sidak test). The effects of repetitive peripheral stimulation on the amplitude of motor evoked potentials were evaluated in each group of rats (controls, administration of Ringer's, b78, or b96.11) using the Student t-test. We also used the analysis of variance to compare the groups. The mean F/mean M response ratio was compared using the analysis of variance, followed by multiple comparison procedure (as above).

## Results

### Patients' antibodies CA/SPS/Ctrl

#### Metabolites in cerebellar nuclei

**Figure 2:**
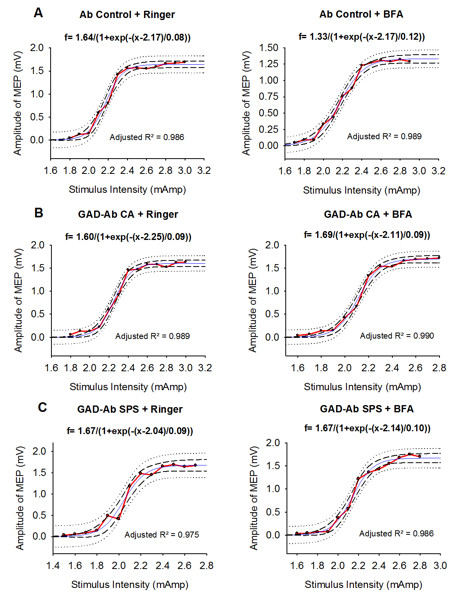
**Sigmoidal fitting of recruitment curves of motor evoked potentials (MEP)**. Upper panels: Ab Ctrl, middle panels: Ab CA, lower panels: Ab SPS. Left panels: Ringer's. Right panels: BFA. Amplitudes are expressed in mV. Dash: 95% prediction band, dotted: 95% confidence band, p < 0.01 for each side. R^2 ^value > 0.970 (p < 0.005).

Ab SPS increased dramatically the extracellular glutamate level during intra-nuclear infusion of NMDA, as compared to Ab CA and Ab Ctrl (p < 0.001) (Figure 2). By contrast, there was no difference in glucose turnover or lactate/pyruvate ratios, indicating that the antibodies did not impair the energy metabolism in cerebellar nuclei. Interestingly, the glycerol turnover was markedly reduced with Ab CA as compared to Ab Ctrl (p < 0.001) and Ab SPS (p < 0.001). These results suggested that Ab CA and Ab SPS exerted different effects upon GAD65 activity and cellular membranes turnover.

#### Sigmoidal fitting

An example of the sigmoidal fitting of recruitment curves of MEPs is illustrated in Figure 2. In each case (Ab Ctrl, Ab CA, and Ab SPS), a significant sigmoidal fit was obtained.

#### Cerebello-cortical inhibition

**Figure 3:**
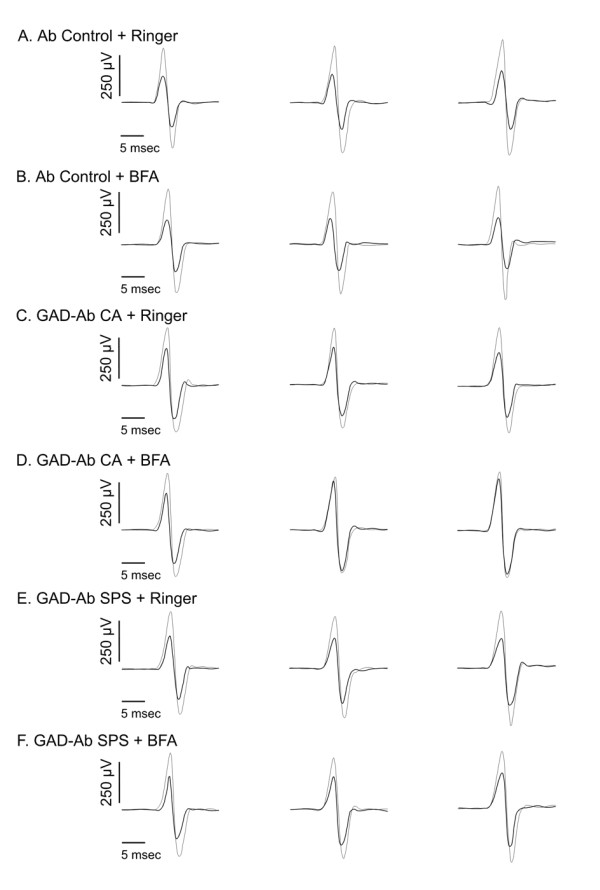
**Cerebello-cortical inhibition**. Thin trace: no conditioning stimulus; thick trace: test stimulus with conditioning stimulus. Left panels: baseline, middle panels: high-frequency, right panels: trains of tDCS. Microinjection of Ab Ctrl in A and B; Ab CA in C and D; Ab SPS in E and F.

**Figure 4:**
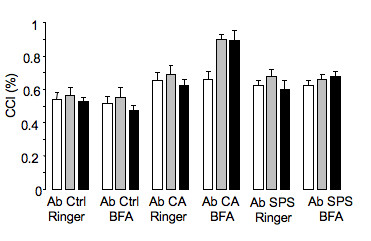
**Cerebello-cortical inhibition (CCI)**. Condition 1: baseline, Condition 2: high-frequency stimulation of the cerebellar cortex, Condition 3: trains of tDCS. BFA and high-frequency stimulation of the cerebellar cortex in rats injected with Ab CA (triangles), Ab Ctrl (circles), and Ab SPS (squares). Values are mean +/- SD. *: p < 0.05.

With Ab CA, cerebello-cortical inhibition remained unchanged following infusion of Ringer's, but decreased following administration of BFA and high-frequency stimulation of the cerebellar cortex (Figure 3A-F). This phenomenon was not observed with Ab Ctrl, or Ab SPS. Ratios of amplitudes of conditioned responses divided by amplitudes of unconditioned responses for the different groups of rats are illustrated (Figure 4).

#### Effects of tDCS

**Figure 5:**
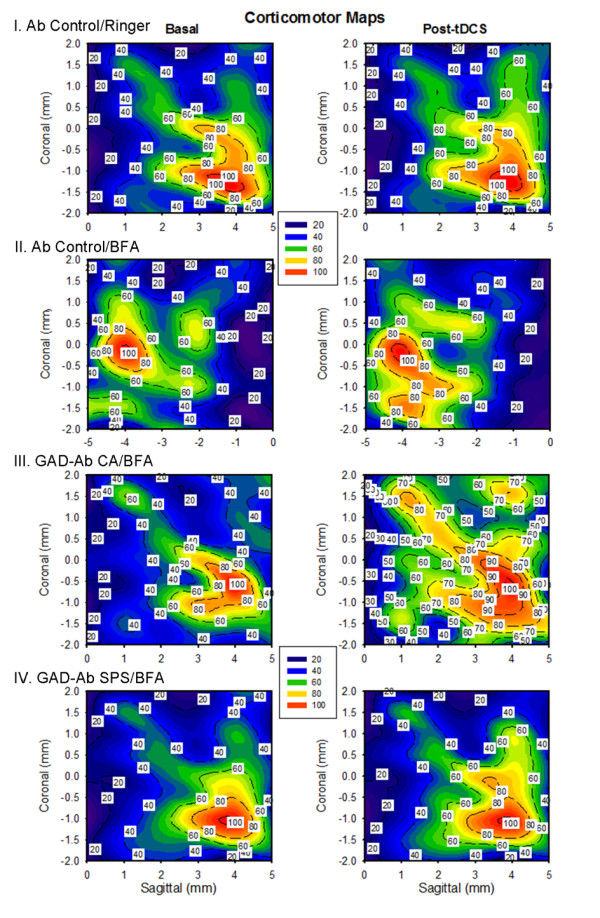
**Motor maps (contour plots and color code)**. X-axis: sagittal coordinates, Y-axis coronal coordinates, Z-axis: corticomotor responses recorded in the contralateral gastrocnemius muscle. So-called "hot spots" (red) are identified. Coordinates of stimulation are established using the stereotactic frame. Maximal responses set at 100% on each side. Left panels: basal, right panels: high-frequency and trains of tDCS.

Ab CA induced marked impairment of corticomotor excitability following the administration of high-frequency cerebellar stimulation (Figure 5). Indeed, trains of tDCS unraveled a spreading of high-level excitability areas of the motor cortex contralaterally to the administration of Ab CA. This phenomenon was not observed with Ab SPS or with Ab Ctrl.

#### Skeletal muscle/spinal cord function

**Figure 6:**
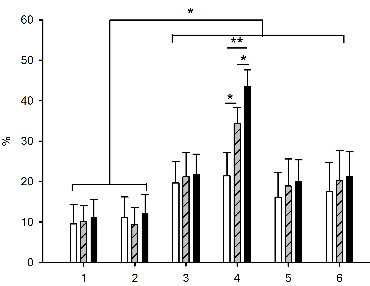
**Mean F/M ratios**. Group 1: Ab Ctrl+Ringer's, left side; Group 2: Ab Ctrl+BFA, right side; Group 3: Ab CA+Ringer's, left side; Group 4: Ab CA+BFA, right side; Group 5: Ab SPS+Ringer's, left side; Group 6: Ab SPS+BFA, right side. White bars: baseline, dash: high-frequency stimulation, black: tDCS. Means (+/- SD) are illustrated. Ratios are in %. *: p < 0.05, **: p < 0.01.

BFA did not impair the Mean F/Mean M ratios in rats receiving Ab Ctrl, including when the high-frequency stimulation of the cerebellum or trains of tDCS were applied (Figure 6). In contrast, the administration of Ab CA and Ab SPS increased the Mean F/M ratios. Mean F/M ratios increased markedly following high-frequency stimulation of the cerebellar cortex after administration of Ab CA and BFA. The increase was even stronger following tDCS of the motor cortex.

### GAD65 enzyme inhibition serum and GAD65Ab epitope pattern

**Figure 7:**
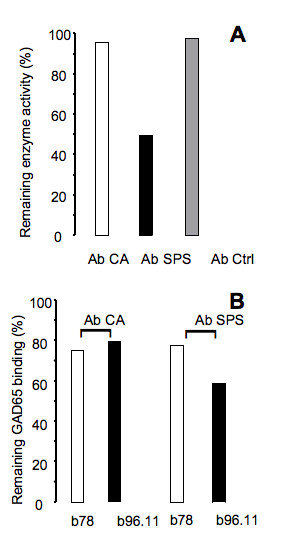
**GAD65 enzyme inhibition and epitope specificity of Ab SPS, Ab CA, and Ab Ctrl**. A: Enzyme activity in the presence of Ab SPS, Ab CA, and Ab Ctrl is reported as percent activity with enzyme activity in the absence of Ab set as 100%. B: Binding of Ab SPS and Ab CA to radiolabeled GAD65 in the presence of rFab b78 and b96.11 is reported as percent binding with uncompeted binding set at 100%.

Inhibition of GAD65 enzyme activity by Ab CA, Ab SPS, and Ab Ctrl was tested (Figure 7A). GAD65 enzyme activity was significantly inhibited by Ab SPS only (50%), while Ab CA had no effect on the enzyme activity. GAD65 binding by Ab SPS was significantly reduced in the presence of rFab b96.11 (59% of remaining binding) and b78 (77% of remaining binding), while GAD65 binding by Ab CA was reduced only in the presence of rFab b78 (75% of remaining binding).

### Monoclonal Ab b78 and b96.11

#### Metabolites in cerebellar nuclei

**Figure 8:**
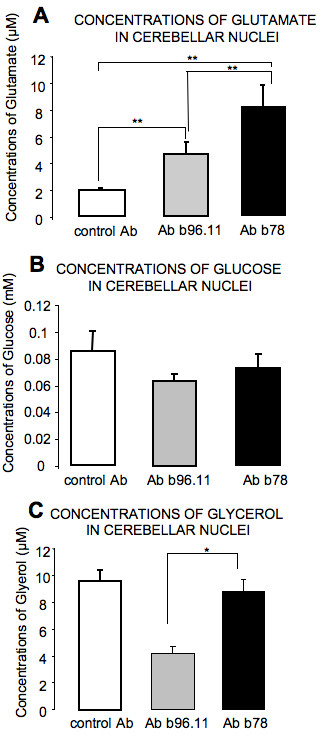
**Concentrations of glutamate, glucose, and glycerol in cerebellar nuclei**. A: Concentrations of glutamate in control rats, rats receiving b96.11, and b78. Values are mean +/- SD. **: p < 0.01. B: glucose concentrations in cerebellar nuclei during NMDA challenge following infusion of b96.11 and b78. C: glycerol concentrations following administration of b96.11 and b78. Values are mean +/- SEM for glucose, median +/- SEM for glycerol. *: p < 0.05.

b78 and b96.11 increased the glutamate concentrations in cerebellar nuclei (Figure 8A). The increase was significantly more pronounced with b78 as compared to b96.11 (p = 0.003). The effects of both antibodies on glucose levels in cerebellar nuclei were similar (Figure 8B). However, glycerol levels were significantly lower with b96.11 as compared to b78 (p = 0.021) (Figure 7C).

#### Cerebello-cortical inhibition

Infusion of rats (n = 2) with b78 after administration of BFA and high-frequency stimulation of the cerebellar cortex resulted in a markedly impaired response (CCI of 0.94%), while rats treated with b96.11 showed CCI comparable to control animals (0.75%) (data not shown).

#### Potentiation of the corticomotor response

**Figure 9:**
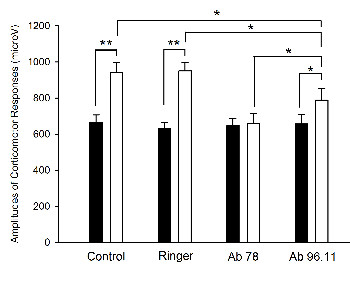
**Modulation of the excitability of the motor cortex**. Control: no cerebellar intervention; Ringer: intra-cerebellar administration of Ringer's; b78: administration of b78; b96.11 administration of b96.11. Recordings in gastrocnemius muscle, before (black columns) and after ipsilateral intra-cerebellar microinjections (white columns). *: p < 0.05; **: p < 0.01.

Peripheral repetitive stimulation increased the amplitudes of MEPs in animals without injection, with Ringer's, and with b96.11 infusion, but not with b78 infusion (p < 0.001, p < 0.001, p = 0.019, and p = 0.37, respectively) (Figure 9). The enhancement of the corticomotor response associated with repetitive stimulation of the sciatic nerve was decreased with b96.11 as compared to the enhancement in the control group (p = 0.017) and absent with b78 as compared to the control group (p < 0.001). Amplitudes of corticomotor potentials after peripheral repetitive stimulation were significantly higher in rats injected with b96.11 as compared to rats injected with b78 (p = 0.024).

#### Effects of tDCS

**Figure 10:**
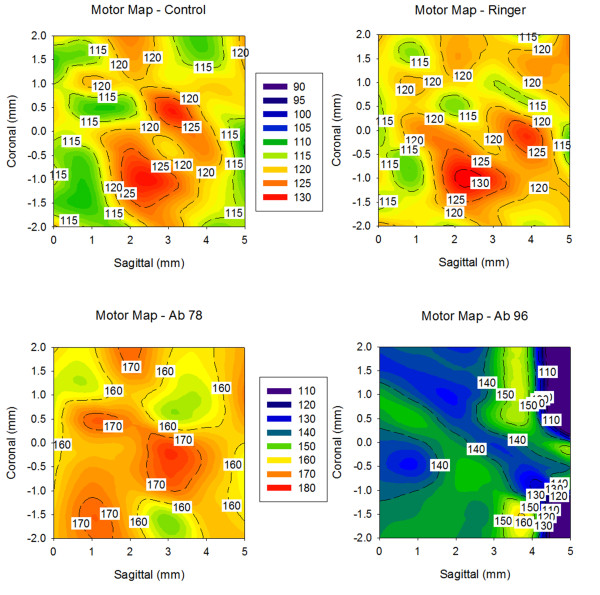
**Effects of tDCS on the amplitudes of motor evoked potentials (contour plots)**. X-axis: sagittal coordinates, Y-axis coronal coordinates, Z-axis: intensities of corticomotor responses. Coordinates of stimulation are established using the stereotactic frame. Responses are expressed as % of baseline. Upper left: control rat; upper right: Ringer's; lower left: b78; lower right: b96.11.

tDCS induced a diffuse and marked increase of the intensity of corticomotor responses following administration of b78, as compared to administration of Ringer's. The increase was milder with b96.11 (Figure 10).

#### F/M Ratios

**Figure 11:**
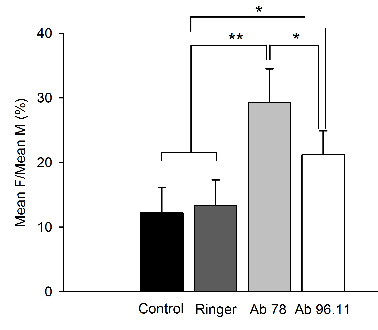
**F/M ratios**. Controls: (black column), Ringer's administration: (dark grey column), b78: (grey column), b96.11: (white column). Values obtained in left plantaris following stimulation of the plantar nerve. Means (+/- SD) are illustrated. Ratios are expressed in %. *: p < 0.05, **: p < 0.01.

A highly significant increase of F/M ratios with b78 or b96.11 as compared to control rats with no injection (p < 0.001 and 0.022, respectively) (Figure 11) and rats with Ringer's administration (p < 0.001 and 0.015, respectively) was found. The values observed after injection with b78 were significantly higher than those for b96.11 (p = 0.011).

The effects of the different polyclonal and monoclonal antibodies on the various parameters are summarized in Table 2.

**Table 2:** Summary of effects of polyclonal and monoclonal antibodies on parameters measured in this study.

	Glutamate levels	Glycerol turnover	Cerebello-cortical inhibition	Corticomotor response	F/M ratio	Enzyme inhibition	Epitope pattern
Ab SPS	Increase	No effect	No effect	No effect	Increase	Inhibition	Inhibition by: b78, b96.11
Ab CA	No effect	Reduction	Decrease	Impairment	Increase	No effect	Inhibition by: B78
Ab Ctrl	No effect	No effect	No effect	No effect	No effect	No effect	No effect
B96.11	Increase	No effect	Decrease	Impairment	Increase	No effect	Inhibition by: B96.11
B78	Strong increase	Reduction	Strong decrease	Strong impairment	Strong increase	Inhibition	Inhibition by: B78

## Discussion

The main finding of this study is that GAD65-Ab induced degree of impairment of neurological functions in vivo depends on the epitope specificity of the respective GAD65-Ab. To study the effect of disease- and epitope-specific GAD65-Ab on neurological functions in vivo, we utilized purified IgG from the serum of a CA and a SPS patient, and two GAD65-specific monoclonal antibodies, of which only one (b78) inhibited the enzyme activity of GAD65. GAD65Ab present in Ab SPS and Ab CA showed different GAD65Ab binding pattern, while also demonstrating some overlap in GAD65Ab epitopes. This finding is in agreement that GAD65Ab in patients are oligo- or polyclonal and thus recognize a wide range of epitopes. The differences in epitope specificities between the two sera was clearly demonstrated by the strong inhibitory effect of Ab SPS on GAD65 enzyme activity, a characteristic often observed for SPS patients [5,6,25], while Ab CA had no effect on the enzyme activity.

In cell and tissue culture systems, IgG from patients with neurological syndromes and GAD65-Ab suppress GABA release, [40,41] indicating that they may change the balance between glutamate and GABA and cause glutamate excitotoxicity. To test this hypothesis in vivo, we studied the effect of GAD65-Ab on the glutamate-pathway by microdialysis. As expected, infusion of NMDA in the cerebellar nuclei reduced the extracellular glutamate concentration in control animals, while glutamate levels were significantly higher in rats injected with b78 and Ab SPS. The GAD65 enzyme-inhibiting monoclonal antibody b78 also impaired glycerol turnover, induced changes of corticomotor excitability, and impaired spinal cord function. These results suggest that inhibition of GAD65 enzyme activity could be responsible for neurochemical and neurophysiological deficits as already suggested [12]. Inhibition of GAD65 enzyme activity could induce the accumulation of the substrate glutamate, leading to glutamate excitotoxicity. However, another potential mechanism for b78 could be through the interference with GAD65-mediated exocytosis of GABA-containing vesicles. Indeed, many arguments suggest that GAD65 may play a specific role in the control of the synaptic release of GABA. GAD65 is localized to the nerve termini and is reversibly bound to the membrane of synaptic vesicles [42-44]. Interestingly, studies in GAD65 knock out mice showed that both the quantal size and frequency of GABA-mediated miniature inhibitory postsynaptic currents (IPSCs) appear to be normal in these knock out mice, but that GABA is reduced during sustained stimulation [42,43,45], an observation in accordance with the effects of our antibodies. Thus binding of GAD65 by antibodies may interfere with the transport and docking of the GABA-containing vesicles. Our observation of a reduced membrane turnover by b78 and Ab CA supports this hypothesis. While the exact function of GAD65 in the synaptic release of GABA remains unclear, our data strongly indicate that GAD65 plays a major role in this process.

Interestingly, a recent study demonstrated that antibodies to amphiphysin, which are associated with paraneoplastic SPS, can access neurons and inhibit neurotransmitter release, in vitro and in vivo, with a mechanism very similar with our antibodies effects [17]. While our results are in agreement with the hypothesis of a GAD65Ab-mediated pathogenesis, we cannot exclude that the patients' sera also contain antibodies directed to GABA(A)-receptor-associated protein, as have been reported in SPS patients [46]. However, the correspondence between effects produced by the sera and the purified monoclonal antibodies support our notion of a pathogenic role of GAD65Ab.

Another interesting point of our results was that intracerebellar injection of b78 abolished the adaptation of the motor cortex. The cerebellum plays a key role in the increase of the cortical motor response normally induced by repeated somatosensory stimulation in rodents [14]. The modulation of this response is considered as the first step for learning in the paradigm of sustained peripheral stimulation [13,15,39]. The effect of b78 intracerebellar injection shows that the adaptation of the cortical motor response following a repetitive somatosensory stimulation is dependent on the GABAeric Purkinje-cerebellar nuclei synapses. The increase in F/M ratios was significantly greater with b78, which inhibits GAD65 enzyme activity, as compared to b96.11, which has no effect on GAD65 enzyme activity, further suggesting the dependence of this process on GAD65 function and GABA production and/or release. We showed previously that IgG from GAD65-Ab positive individuals without CNS involvement were ineffective in changing the F/M ratios [12]. Our results provide an explanation for the distinct phenotypes presented by GAD65-Ab from SPS patients and CA patients. Indeed, we observed that glutamate levels were higher in rats treated with Ab SPS, whereas the turnover of membranes was impaired by Ab CA. We hypothesize that the latter is caused by an inhibition of membrane turnover and we were able to confirm this hypothesis through the use of BFA and a high-frequency stimulation procedure. Our data show that some GAD65-Ab induce a vulnerability to high-frequency stimulation in terms of dependence of endosomal recycling. Experiments with BFA confirmed a BFA-dependence in the presence of Ab CA, indicating that these antibodies induced a state of dependence to compensatory mechanisms of exocytosis. This could also explain why symptoms of SPS may improve with immunotherapy and IgG-depleting strategies, while symptoms of cerebellar dysfunction rarely improve [20-22]. Indeed, the cascade of events induced by antibodies differs in both conditions.

Finally, our results suggest new strategies to study neuronal loops controlling corticomoto-neuronal excitability. Continuous or intermittent anodal tDCS induces a polarity-dependent modulation of brain activity. The functional modifications observed with anodal tDCS are site-specific. Anodal stimulation increases cortical excitability, by reducing intra-cortical inhibition and by augmenting facilitation [47]. The activity of the motor cortex is greatly dependent on the balance between excitatory and inhibitory influences over the network of cortical connections. The cerebellothalamocortical pathway is the most probable candidate for providing the input for gating the information flow. Cerebellar information is guided to the primary motor cortex via the ventro-lateral thalamic group, which projects mainly to layers IV and V [48]. Through this channel, inputs can adjust the features of the circuitry of the motor cortex in various contexts. We show that monoclonal GAD65-Ab impaired the excitability of the motor cortex and that tDCS enhances this perturbation, particularly with b78. This is a new method to explore the consequences of GAD65-Ab upon corticomotoneuronal excitability.

## Conclusions

Our previous results strongly supported the hypothesis that antibodies are directly involved in the pathogenesis of SPS and CA [12]. We now show that GAD65-Ab could be directly responsible for this defect and that the epitope specificity of the GAD65-Ab is crucial to explain the development of different neurological syndromes. Further work will be necessary to understand how IgG can be internalized by neurons and how the GAD65-Ab can modify production and synaptic release of GABA.

## Competing interests

The authors declare that they have no competing interests.

## Authors' contributions

M-UM participated in the design, carried out the neurological analyses. CSH carried out the enzyme assays and epitope mapping experiments. VR managed the patient's sera and CSF and purified the IgG. JH conceived of the study and participated in its design. All the authors have contributed to the interpretation of the results and have participated in the draft of the manuscript. All authors read and approved the final manuscript.
